# Polymorphism of the Fractalkine Receptor CX3CR1 and Systemic Sclerosis-associated Pulmonary Arterial Hypertension

**DOI:** 10.1080/17402520500303297

**Published:** 2005-12

**Authors:** Bianca Marasini, Roberta Cossutta, Carlo Selmi, Maria Rosa Pozzi, Marco Gardinali, Marco Massarotti, Maddalena Erario, Lodovica Battaglioli, Maria Luisa Biondi

**Affiliations:** 1 Rheumatology Unit Department of Medicine Surgery and Dentistry, Humanitas Clinical Institute University of Milan, Rozzano Milan Italy; 2 Division of Internal Medicine San Paolo School of Medicine University of Milan Italy; 3 Division of Rheumatology, Allergy, Clinical Immunology University of California Davis CA USA; 4 Department of Medicine S. Gerardo Hospital Monza Italy; 5 Clinical Chemistry Laboratory S. Paolo Hospital Milan Italy

## Abstract

Fractalkine (FKN) and its receptor CX3CR1 are critical mediators in the vascular and tissue damage of several chronic diseases, including systemic sclerosis (SSc) and pulmonary arterial hypertension (PAH). Interestingly, the V249I and T280M genetic polymorphisms influence CX3CR1 expression and function. We investigated whether these polymorphisms are associated with PAH secondary to SSc. CX3CR1 genotypes were analyzed by PCR and sequencing in 76 patients with limited SSc and 204 healthy controls. PAH was defined by colorDoppler echocardiography. Homozygosity for 249II as well as the combined presence of 249II and 280MM were significantly more frequent in patients with SSc compared to controls (17 vs 6%, *p* =  0.0034 and 5 vs 1%, *p* =  0.0027, respectively). The 249I and 280M alleles were associated with PAH (odd ratio [OR] 2.2, 95% confidence interval [CI] 1.01-4.75, *p* =  0.028 and OR 7.37, 95%CI: 2.45-24.60, *p* =  0.0001, respectively). In conclusion, the increased frequencies of 249I and 280M CX3CR1 alleles in a subgroup of patients with SSc-associated PAH suggest a role for the fractalkine system in the pathogenesis of this condition. Further, the 249I allele might be associated with susceptibility to SSc.


*Clinical & Developmental Immunology*, December 2005; 12(4): 275–279

Taylor & Francis

Taylor & Francis Group

### Polymorphism of the fractalkine receptor CX3CR1 and systemic sclerosis-associated pulmonary arterial hypertension

BIANCA MARASINI[1], ROBERTA COSSUTTA[1], CARLO SELMI[2,3], MARIA ROSA POZZI[4], MARCO GARDINALI[4], MARCO MASSAROTTI[1], MADDALENA ERARIO[5], LODOVICA BATTAGLIOLI[5], & MARIA LUISA BIONDI[5]

[1]*Rheumatology Unit, Department of Medicine, Surgery and Dentistry, Humanitas Clinical Institute, University of Milan, Rozzano, Milan, Italy,* [2]*Division of Internal Medicine, San Paolo School of Medicine, University of Milan, Italy,* [3]*Division of Rheumatology, Allergy, Clinical Immunology, University of California, Davis, CA, USA,* [4]*Department of Medicine, S. Gerardo Hospital, Monza, Italy, and* [5]*Clinical Chemistry Laboratory, S. Paolo Hospital, Milan, Italy*

**Abstract**

Fractalkine (FKN) and its receptor CX3CR1 are critical mediators in the vascular and tissue damage of several chronic diseases, including systemic sclerosis (SSc) and pulmonary arterial hypertension (PAH). Interestingly, the V249I and T280M genetic polymorphisms influence CX3CR1 expression and function. We investigated whether these polymorphisms are associated with PAH secondary to SSc. CX3CR1 genotypes were analyzed by PCR and sequencing in 76 patients with limited SSc and 204 healthy controls. PAH was defined by colorDoppler echocardiography. Homozygosity for 249II as well as the combined presence of 249II and 280MM were significantly more frequent in patients with SSc compared to controls (17 vs 6%, $p = 0.0034$ and 5 vs 1%, $p = 0.0027$, respectively). The 249I and 280M alleles were associated with PAH (odd ratio [OR] 2.2, 95% confidence interval [CI] 1.01–4.75, $p = 0.028$ and OR 7.37, 95%CI: 2.45–24.60, $p = 0.0001$, respectively). In conclusion, the increased frequencies of 249I and 280M CX3CR1 alleles in a subgroup of patients with SSc-associated PAH suggest a role for the fractalkine system in the pathogenesis of this condition. Further, the 249I allele might be associated with susceptibility to SSc.

**Keywords:** *Fractalkine, genetics, pulmonary hypertension, scleroderma*

### Introduction

Systemic sclerosis (SSc) is an autoimmune disease of unknown etiology characterized by microvascular changes and progressive skin and visceral organ fibrosis. Vascular alterations and cellular infiltrations in target tissues are early features in the course of the disease (Prescott et al. 1992, Kraling et al. 1995) while pulmonary arterial hypertension (PAH) is a common complication of SSc, often with poor prognosis (Brundage 1990, MacGregor et al. 2001). Recent evidence indicates that chemokines produced by endothelial cells induce leukocyte transvascular migration ultimately leading to tissue damage in several chronic diseases, including SSc (Atamas and White 2003, Hussein et al. 2005). Fractalkine (FKN) is a recently discovered chemokine, that acts as adhesion molecule via its specific receptor CX3CR1 on leukocytes when in its membrane-bound form, but also as chemotaxic stimulus when in its soluble form after proteolytic cleavage (Bazan et al. 1997). Importantly, an abnormal expression or function of the FKN/CX3CR1 system is believed to be involved in inflammatory conditions leading to vascular and tissue damage (Ancuta et al. 2003, Umehara et al. 2004).

Two coding CX3CR1 single nucleotide polymorphisms (V249I, T280M) have been recently described (Faure et al. 2000) with the 249I allele associated with a reduced prevalence of atherosclerosis (McDermott et al. 2001, Moatti et al. 2001, Ghilardi et al. 2004) possibly secondary to a decreased affinity of FKN to its receptor. Since a role for FKN in PAH (Balabanian et al. 2002) and

Correspondence: B. Marasini, Humanitas Clinical Institute, Via Manzoni 56, 20089, Rozzano, Milan, Italy. Tel: 39 02 82244075, Fax: 39 02 82242298. E-mail: bianca.marasini@humanitas.it

ISSN 1740-2522 print/ISSN 1740-2530 online © 2005 Taylor & Francis
DOI: 10.1080/17402520500303297

Table I. Odd ratios for genotypes were calculated with V/I + V/V vs I/I and T/M + T/T vs M/M.

| | Patients (No. 76) | Controls (No. 204) | OR (95% CI) | P |
|---|---|---|---|---|
| V249I | | | | |
| V/V | 42 (55%) | 108 (53%) | | |
| V/I | 21 (28%) | 84 (41%) | | |
| I/I | 13 (17%) | 12 (6%) | 3.30 (1.30-8.32) | 0.0034 |
| I allele frequency | 0.31 | 0.27 | 1.24 (0.80-1.90) | 0.29 |
| T280M | | | | |
| T/T | 56 (74%) | 142 (70%) | | |
| T/M | 16 (21%) | 58 (28%) | | |
| M/M | 4 (5%) | 4 (2%) | 1.22 (0.65-2.33) | 0.50 |
| M allele frequency | 0.16 | 0.16 | 1.02 (0.60-1.79) | 0.91 |

SSc (Hasegawa et al. 2005) has been suggested and prompted by the need to identify patients with SSc at risk for PAH, we studied whether genetic variability of the FKN/CX3CR1 system could be involved in SSc-associated PAH.

### Materials and methods

### *Study population*

Seventy six patients (74 females, mean ± standard deviation age 62 ± 12 years) affected with limited SSc according to the classification of LeRoy et al. (1988) were enrolled in the study. All patients fullfilled the American College of Rheumatology criteria for SSc diagnosis (Preliminary criteria for the classification of systemic sclerosis (scleroderma). Subcommittee for scleroderma criteria of the American Rheumatism Association Diagnostic and Therapeutic Criteria Committee 1980), and underwent colorDoppler echocardiography for the definition of PAH. A pulmonary artery systolic pressure (PASP) $>35$ mm Hg was used to define PAH (Schachna et al. 2003). Sex-matched healthy subjects with no evidence of cardiovascular diseases ($n = 204$) served as controls.

### *DNA extraction and CX3CR1 genotyping*

Following informed consent, whole blood was obtained from all SSc cases and controls. DNA extraction and CX3CR1 genotyping were performed as previously described (Ghilardi et al. 2004).

### *Statistical analysis*

Differences in genotype and allele frequencies between groups were compared using the chi-square test. Odds ratios (OR) (approximate relative risk) and 95% confidence interval (CI) were calculated for the associations of the V249I and T280M genotypes with phenotypes. All analyses were two-tailed and P values lower than 0.05 were considered as statistically significant. Stata Statistical Software (Stata Corporation, College Station, TX) was used for statistical analyses.

### Results

The genotype distributions for both I249V and T280M polymorphisms was in accordance with the Hardy-Weinberg law among control subjects but not in patients with SSc where excesses of 249II ($p = 0.002$) and 280MM ($p = 0.018$) were observed.

The adjusted ORs associated with the presence of 249I and the 280M alleles were 3.30 (95%CI: 1.30–8.32, $p = 0.0034$) and 1.22 (95%CI: 0.65–2.33, $p = 0.50$), respectively (Table I).

Six genotype combinations were possible when I249V and T280M genotypes were combined (Table II). Combinations 1 (249VV and 280TT) and 6 (249VI and 280TM) were the most common in both populations while combination 3 (249II and

Table II. Combined genotype frequencies of the V249I and T280M polymorphisms of the CX3CR1 in systemic sclerosis patients and controls.

| Combined genotype | V249I | T280M | Patients No (%) | Controls No (%) | P |
|---|---|---|---|---|---|
| 1 | VV | TT | 42 (56) | 104 (51) | Ns |
| 2 | II | TT | 5 (6) | 4 (2) | 0.05 |
| 3 | II | MM | 4 (5) | 2 (1) | 0.027 |
| 4 | II | TM | 4 (5) | 6 (3) | Ns |
| 5 | VI | TT | 9 (12) | 36 (18) | Ns |
| 6 | VI | TM | 12 (16) | 52 (25) | 0.08 |

Global haplotype effect $\chi^2 = 12.85$, $p = 0.027$.

Table III. **3.** Odds ratios for genotypes were calculated with V/I + V/V vs I/I and T/M + T/T vs M/M.

| | PASP <35 mm Hg *N*=50 (*n*) | PASP >35 mm Hg, *N*=26 (*n*) | OR (95%CI) | *P* |
|---|---|---|---|---|
| V/V | 30 (60%) | 12 (47%) | | |
| V/I | 15 (30%) | 6 (23%) | | |
| I/I | 5 (10%) | 8 (30%) | 4 (0.97–17.43) | 0.022 |
| I allele frequency | 0.25 | 0.42 | 2.2 (1.01–4.75) | 0.028 |
| T/T | 44 (88%) | 12 (46%) | | |
| T/M | 6 (12%) | 10 (38%) | | |
| M/M | 0 (0%) | 4 (15%) | | 0.004 |
| M allele frequency | 0.06 | 0.35 | 7.37 (2.45–24.6) | 0.0001 |

280MM) was found significantly more frequently in patients with SSc compared to controls ($p = 0.027$).

Table III shows the relationship between CX3CR1 polymorphisms and the presence of PAH. The 249II genotype was found in 8/26 (30%) patients with SSc-associated PAH and 5/50 (10%) of patients with PASP values <35 mmHg and such genotype was, therefore, significantly associated with PAH (OR 4, 95% CI 0.97–17.43; $p = 0.022$). Accordingly, the 249I allele frequency was significantly higher in patients with PAH (0.42 vs. 0.25 in patients without PAH, OR 2.2; 95% CI 1.01–4.75, $p = 0.028$). Finally, the 380MM genotype was observed in 4/26 patients with PAH and 0/50 patients with normal PASP values ($p = 0.004$).

### Discussion

SSc is an enigmatic autoimmune disease burdened by several types of serious complications. Among these, isolated PAH is more frequent in the limited form of SSc (Koh et al. 1996) and its prognosis is often poor, particularly because of elusive and late diagnosis and despite the available novel treatments (Badesch et al. 2004; McLaughlin et al. 2004), thus representing the most frequent cause of death in limited SSc (MacGregor et al. 2001). For these reasons, a non-invasive marker to specifically identify patients who are more prone to developing PAH should be a primary goal of SSc research.

There is increasing evidence for a role of chemokines in initiating and perpetuating endothelial cell activation as one of the key events leading to tissue damage in several vascular diseases, including SSc (Fujii et al. 2004). A link between endothelial cell dysfunction and the early cellular infiltration across the vessel wall into SSc-targeted tissues has been demonstrated (Prescott et al. 1992) while other reports have shown an upregulated leukocyte trafficking into SSc connective tissues (Rudnicka et al. 1992). Importantly, FKN is one of the most potent molecules that regulate inflammatory cell trafficking through the endothelium (Fong et al. 1998) and recent findings suggest a possible role for the FKN/CX3CR1 system in SSc pathogenesis (Hasegawa et al. 2005). Similar to SSc, leukocyte trafficking with extravasation in response to chemokines plays a role in PAH pathogenesis (Dorfmuller et al. 2002) and pulmonary perivascular inflammatory infiltrates of patients with SSc-associated PAH (Cool et al. 1997) indicate a possible role for vascular inflammation also in SSc-related PAH. Further, Balabanian et al. have suggested a major role for FKN/CX3CR1 in PAH pathogenesis, since they demonstrated that T cells from patients with PAH have upregulated CX3CR1, alongside with high FKN plasma concentrations, FKN hyper-expression in lung parenchyma and pulmonary artery endothelial cells.

For these reasons, we have performed a case-control association study to determine if CX3CR1 genetic polymorphisms were associated with SSc and SSc-associated PAH. Our data indicate that 429I allele and 480M allele frequencies are significantly increased in SSc patients with PAH, thus supporting the observations of Balabanian et al. and suggesting that FKN/CX3CR1 system might play a role also in SSc-associated PAH pathogenesis, albeit in a small subgroup of patients with SSc. On the other hand, we observed that 249II genotype is associated with susceptibility to SSc.

Interestingly, CX3CR1 polymorphisms were shown to exert a protective effect against coronary and carotid atherosclerosis (McDermott et al. 2001; Moatti et al. 2001; Ghilardi et al. 2004), thus apparently conflicting with our findings. The reasons for this apparent discrepancy might be rather complex, similar to the FKN/CX3CR1 pathway (Daoudi et al. 2004) and several issues need to be addressed. First, the protective effect of 249I on coronary arteries has been reported to be associated with a lower number of cell surface binding sites, although highly variable among individuals with the same genotype (Moatti et al. 2001) while the resulting lower FKN affinity has been recently questioned, being the 249I homozygous genotype associated with enhanced adhesiveness (Daoudi et al. 2004). Also, we note that a clear definition of the specific polymorphism contribution is difficult since they are in linkage disequilibrium. Second, differences related to the characteristics of specific tissue vessels cannot be overlooked. In fact, the same CX3XR1 genotypes were not protective against ischaemic stroke (Hattori et al. 2005; Lavergne et al. 2005), nor against peripheral arterial disease (Gugl et al. 2003), but in the same patients I249 and M280 alleles were associated both with an increased risk of brain infarction and a reduced frequency of cardiovascular history (Lavergne et al. 2005). Third, we cannot rule out at present that other aspects of the FKN/CX3CR1 system might be interplaying. Lucas et al. reported that FKN has a role in vascular remodelling through the recruitment of smooth muscle cells, a prominent histological feature of both PAH and SSc. Further, remodelling is regulated by oxygen levels through the regulation of the expression of vasoactive substances and smooth muscle mitogens (Faller 1999) and the interference with mechanisms of angiogenesis (Koch 2000). Interestingly, hypoxia can inhibit FKN expression (Yamashita et al. 2003) while also functioning as an angiogenic mediator (Volin et al. 2001) thus possibly contributing either to the altered angiogenesis and the ischaemic changes of SSc vasculopathy (LeRoy 1996) or to the pulmonary arterial lesions of PAH. Fourth, the mechanisms underlying atherosclerosis formation are likely different from those leading to the vascular remodelling observed in SSc and PAH, where pathological resemblance (Tuder et al. 1994; Cool et al. 1997) might suggest a similar pathogenesis of the tissue injury.

We failed to identify an association between CX3CR1 polymorphisms and pulmonary fibrosis (data not shown); Hasagawa et al. reported significantly elevated plasma FKN levels in patients with SSc-associated pulmonary fibrosis but not in those with PAH. This discrepancy may be due to the small number of limited SSc, coupled with the well known higher prevalence of pulmonary fibrosis in diffuse SSc (Steen 2003). In our study, the objective of studying PAH made the choice of enrolling patients with limited SSc more appropriate.

Future studies are warranted to confirm the proposed association, as well as to better define the role of the FKN/CX3CR1 system in SSc and other diseases characterized by vascular inflammation and remodelling. In the case of SSc, the search for non-invasive markers of disease progression or severity should be a priority not only because an early diagnosis may improve SSc-associated PAH prognosis, but also because chemokines and their receptors might provide novel therapeutic targets.

### References

Ancuta P, Rao R, et al. 2003. Fractalkine preferentially mediates arrest and migration of CD16+ monocytes. J Exp Med 197:1701–1707.

Atamas SP, White B. 2003. The role of chemokines in the pathogenesis of scleroderma. Curr Opin Rheumatol 15:772–777.

Badesch DB, Abman SH, et al. 2004. Medical therapy for pulmonary hypertension: ACCP evidence-based clinical practice guidelines. Chest 126(1 Suppl):35S–62S.

Balabanian K, Foussat A, et al. 2002. CX(3)C chemokine fractalkine in pulmonary arterial hypertension. Am J Respir Crit Care Med 165:1419–1425.

Bazan JF, Bacon KB, et al. 1997. A new class of membrane-bound chemokine with a CX3C motif. Nature 385:640–644.

Brundage BH. 1990. Pulmonary hypertension in collagen vascular disease. In: Fishman A, editor. The pulmonary circulation. Philadelphia: University of Pennsylvania. p 353–358.

Cool CD, Kennedy D, et al. 1997. Pathogenesis and evolution of plexiform lesions in pulmonary hypertension associated with scleroderma and human immunodeficiency virus infection. Hum Pathol 28:434–442.

Daoudi M, Lavergne E, et al. 2004. Enhanced adhesive capacities of the naturally occurring Ile249-Met280 variant of the chemokine receptor CX3CR1. J Biol Chem 279:19649–19657.

Dorfmuller P, Zarka V, et al. 2002. Chemokine RANTES in severe pulmonary arterial hypertension. Am J Respir Crit Care Med 165:534–539.

Faller DV. 1999. Endothelial cell responses to hypoxic stress. Clin Exp Pharmacol Physiol 26:74–84.

Faure S, Meyer L, et al. 2000. Rapid progression to AIDS in HIV+ individuals with a structural variant of the chemokine receptor CX3CR1. Science 287:2274–2277.

Fong AM, Robinson LA, et al. 1998. Fractalkine and CX3CR1 mediate a novel mechanism of leukocyte capture, firm adhesion, and activation under physiologic flow. J Exp Med 188:1413–1419.

Fujii H, Shimada Y, et al. 2004. Serum levels of a Th1 chemoattractant IP-10 and Th2 chemoattractants, TARC and MDC, are elevated in patients with systemic sclerosis. J Dermatol Sci 35:43–51.

Ghilardi G, Biondi ML, et al. 2004. Internal carotid artery occlusive disease and polymorphisms of fractalkine receptor CX3CR1: A genetic risk factor. Stroke 35:1276–1279.

Gugl A, Renner W, et al. 2003. Two polymorphisms in the fracalkine receptor CX3CR1 are not associated with peripheral arterial disease. Atherosclerosis 166:339–343.

Hasegawa M, Sato S, et al. 2005. Up regulated expression of fractalkine/CX3CL1 and CX3CR1 in patients with systemic sclerosis. Ann Rheum Dis 64:21–28.

Hattori H, Ito D, et al. 2005. T280M and V249I polymorphisms of fractalkine receptor CX3CR1 and ischemic cerebrovascular disease. Neurosci Lett 374:132–135.

Hussein MR, Hassan HI, et al. 2005. Alterations of mononuclear inflammatory cells, CD4/CD8+ T cells, interleukin 1beta, and tumour necrosis factor alpha in the bronchoalveolar lavage fluid, peripheral blood, and skin of patients with systemic sclerosis. J Clin Pathol 58:178–184.

Koch AE. 2000. The role of angiogenesis in rheumatoid arthritis: Recent developments. Ann Rheum Dis 59(Suppl. 1):i65–i71.

Koh ET, Lee P, et al. 1996. Pulmonary hypertension in systemic sclerosis: An analysis of 17 patients. Br J Rheumatol 35:989–993.

Kraling BM, Maul GG, et al. 1995. Mononuclear cellular infiltrates in clinically involved skin from patients with systemic sclerosis of recent onset predominantly consist of monocytes/macrophages. Pathobiology 63:48–56.

Lavergne E, Labreuche J, et al. 2005. Adverse associations between CX3CR1 polymorphisms and risk of cardiovascular or cerebrovascular disease. Arterioscler Thromb Vasc Biol 25:847–853.

LeRoy EC. 1996. Systemic sclerosis. A vascular perspective. Rheum Dis Clin North Am 22:675–694.

MacGregor AJ, Canavan R, et al. 2001. Pulmonary hypertension in systemic sclerosis: Risk factors for progression and consequences for survival. Rheumatology (Oxf.) 40:453–459.

McDermott DH, Halcox JP, et al. 2001. Association between polymorphism in the chemokine receptor CX3CR1 and coronary vascular endothelial dysfunction and atherosclerosis. Circ Res 89:401–407.

McLaughlin VV, Presberg KW, et al. 2004. Prognosis of pulmonary arterial hypertension: ACCP evidence-based clinical practice guidelines. Chest 126:78S–92S.

Moatti D, Faure S, et al. 2001. Polymorphism in the fractalkine receptor CX3CR1 as a genetic risk factor for coronary artery disease. Blood 97:1925–1928.

Preliminary criteria for the classification of systemic sclerosis (scleroderma) 1980. Subcommittee for scleroderma criteria of the American Rheumatism Association Diagnostic and Therapeutic Criteria Committee. Arthritis Rheum 23:581–590.

Prescott RJ, Freemont AJ, et al. 1992. Sequential dermal microvascular and perivascular changes in the development of scleroderma. J Pathol 166:255–263.

Rudnicka L, Majewski S, et al. 1992. Adhesion of peripheral blood mononuclear cells to vascular endothelium in patients with systemic sclerosis (scleroderma). Arthritis Rheum 35:771–775.

Schachna L, Wigley FM, et al. 2003. Age and risk of pulmonary arterial hypertension in scleroderma. Chest 124:2098–2104.

Steen V. 2003. Predictors of end stage lung disease in systemic sclerosis. Ann Rheum Dis 62:97–99.

Tuder RM, Groves B, et al. 1994. Exuberant endothelial cell growth and elements of inflammation are present in plexiform lesions of pulmonary hypertension. Am J Pathol 144:275–285.

Umehara H, Bloom ET, et al. 2004. Fractalkine in vascular biology: From basic research to clinical disease. Arterioscler Thromb Vasc Biol 24:34–40.

Volin MV, Woods JM, et al. 2001. Fractalkine: A novel angiogenic chemokine in rheumatoid arthritis. Am J Pathol 159: 1521–1530.

Yamashita K, Imaizumi T, et al. 2003. Effect of hypoxia on the expression of fractalkine in human endothelial cells. Tohoku J Exp Med 200:187–194.

